# Sampling and Pooling Methods for Capturing Herd Level Antibiotic Resistance in Swine Feces using qPCR and CFU Approaches

**DOI:** 10.1371/journal.pone.0131672

**Published:** 2015-06-26

**Authors:** Gunilla Veslemøy Schmidt, Anders Mellerup, Lasse Engbo Christiansen, Marie Ståhl, John Elmerdahl Olsen, Øystein Angen

**Affiliations:** 1 The National Veterinary Institute, Technical University of Denmark, Frederiksberg C, Denmark; 2 Department of Applied Mathematics and Computer Science, Technical University of Denmark, Kongens Lyngby, Denmark; 3 Department of Veterinary Disease Biology, Faculty of Health and Medical Sciences, University of Copenhagen, Frederiksberg C, Denmark; Catalan Institute for Water Research (ICRA), SPAIN

## Abstract

The aim of this article was to define the sampling level and method combination that captures antibiotic resistance at pig herd level utilizing qPCR antibiotic resistance gene quantification and culture-based quantification of antibiotic resistant coliform indicator bacteria. Fourteen qPCR assays for commonly detected antibiotic resistance genes were developed, and used to quantify antibiotic resistance genes in total DNA from swine fecal samples that were obtained using different sampling and pooling methods. In parallel, the number of antibiotic resistant coliform indicator bacteria was determined in the same swine fecal samples. The results showed that the qPCR assays were capable of detecting differences in antibiotic resistance levels in individual animals that the coliform bacteria colony forming units (CFU) could not. Also, the qPCR assays more accurately quantified antibiotic resistance genes when comparing individual sampling and pooling methods. qPCR on pooled samples was found to be a good representative for the general resistance level in a pig herd compared to the coliform CFU counts. It had significantly reduced relative standard deviations compared to coliform CFU counts in the same samples, and therefore differences in antibiotic resistance levels between samples were more readily detected. To our knowledge, this is the first study to describe sampling and pooling methods for qPCR quantification of antibiotic resistance genes in total DNA extracted from swine feces.

## Introduction

Antibiotic resistance in pathogenic bacteria is an increasing problem challenging disease treatment in humans and animals globally [1–3]. It is important to minimize antibiotic use in intensive agricultural practices where widespread antibiotic use is common e.g. in the pig production, as such use can have severe consequences for human health [4]. As resistance is selected for in both commensal and pathogenic bacteria during antibiotic treatment, it is of major concern if commensal bacteria become a reservoir of antibiotic resistance genes for pathogenic bacteria [1,4–6].

Surveillance of the presence of antibiotic resistance in individuals, populations and/or the environment facilitates risk management and may also support correct choice of drug for disease treatment. At present, no good strategy has been published for quantification of resistance levels in animals at herd level. When designing such a strategy, it is important to consider the combination of sampling and analytical methods in order to gain representation of the true resistance level, whilst a feasible time frame and economic resources must be maintained.

The traditional phenotypic methods for surveillance of antibiotic resistance in populations rely on cultures of indicator bacteria such as *Escherichia coli*, *Enterococcus* spp., *Salmonella* spp., and *Campylobacter* spp. However, surveillance by this approach neglects the remaining intestinal microbiota and potentially underestimates the true antibiotic resistance levels in the bacterial community [7]. Therefore, the amount of resources put into the development of nucleic acid-based methods utilizing total bacterial community DNA for antibiotic resistance detection has increased vastly [7,8]. These methods enable detection and quantification of antibiotic resistance, also in the slow-growing, non-cultivable, viable, and non-viable bacteria. Multiple target genes of interest can easily be targeted within the community where bacteria could be sharing resistance determinants thus reflecting the entire gene pool [7]. Real-time PCR (qPCR) is an extremely sensitive method and has been the choice of method for specific and rapid quantification of genes, including genes encoding antibiotic resistance in microbiome samples such as fecal samples and fecal contaminated environments [7,9–11].

The aim of the study was to define the combination of sampling level and quantification method that captures antibiotic resistance at herd level. Herd level antibiotic resistance estimates by qPCR and resistance levels in coliform bacteria were used to analyze swine fecal samples collected at different levels (with and without pooling). The genes included in this study were chosen with disregard to the indicator bacteria as the point was to assess the two methods separately as two approaches to antibiotic resistance surveillance. We recommend either pen floor samples or shoe cover sampling for capturing herd level antibiotic resistance as both sampling types were able to quantify antibiotic resistance genes in swine feces.

## Materials and Methods

### Sample collection

Pig fecal samples were collected from a single feeder pig operation in Denmark at 2 separate time points (sampling 1, during March 2013; sampling 2, during July 2013). The sampling methods were included for the following reasons: Shoe cover sampling is used in the Danish *Salmonella* control program in broiler flocks [12] and it is of interest to investigate whether shoe cover sampling can be applied to pig herds; pen floor sampling is currently used in the Danish *Salmonella* control program in pig herds [13,14]; slurry tank sampling is of interest as the antibiotic resistance levels within the slurry tank may reflect the herd’s levels rendering it a convenient method for herd sampling potentially representing resistance levels for the past 6 months (they are emptied twice yearly). The collected samples, their sizes and how they were pooled prior to analysis are summarized in Table 1. Consent for fecal sample collection was given by the farmer. The sampling was performed by authorized Danish veterinarians and as the sampling did not include invasive handling of animals, no permit from the Animal Inspectorate was required.

**Table 1:** Overview of the samples collected during sampling 1 and sampling 2 including different sampling methods and corresponding laboratory pools at stable and herd levels.

Pool name	Sampling 1	Sampling 2	Samples included in laboratory pool	Number of samples (n)
	1 stable sampled	5 stables sampled		
All animals	+	-	All individual animals from sampling 1 (extraction from rectum)	n = 84
Individual animal pool pen	+	-	Pool of individual animals within each pen (extraction from rectum)	Pen 1 n = 22;pen 2 n = 20;pen 3 n = 22;pen 4 n = 20
Pen floor samples	+	+	Not pooled	Sampling 1 n = 4; sampling 2 n = 20
Pen floor pool stable	+	+	Pool of pen floor samples from pens 1–4 in each stable	Sampling 1 n = 1;sampling 2 n = 5
Pen floor pool herd	-	+	Pool of pen floor samples from each stable (1–5)	n = 1
Shoe cover samples	+	+	Not pooled	Sampling 1 n = 4;sampling 2 n = 10
Shoe cover pool stable	+	+	Pool of the shoe cover samples in each stable	Sampling 1 n = 1;sampling 2 n = 2
Shoe cover pool herd	-	+	Pool of shoe cover samples from each stable (1–5)	n = 1
Slurry tank samples	-	+	Each sample was a pool from 3 depths collected at the same spot (1m, 1.5m, and 2m)	n = 3
Pool slurry	-	+	Pool of slurry tank samples 1–3	n = 1

In order to establish if pooled samples from the floor and shoe covers could be used instead of sampling individual animals, fecal samples were collected from a single stable (stable 1) during sampling 1 (Table 1). In the stable, 4 pens (pens 1–4) were randomly chosen, and within these 4 pens fecal samples from all pigs (pen 1 n = 22, pen 2 n = 19, pen 3 n = 21, pen 4 n = 19; total n = 84) were collected from the rectum. Furthermore, each of the 4 pens was sampled by pooling 5 separate samples of feces from the floor of each pen (pen floor samples, n = 4). Four shoe cover samples were also collected in the same 4 pens. Two people each wore a pair of blue disposable polypropylene shoe covers (SEPA, Hilleroed, Denmark) and the pen area was covered by walking throughout the entire pen in a systematic snake formed pattern thus covering as much of the pen floor as possible. The same shoe covers were repeatedly used in all 4 pens and were removed when walking between pens.

In order to establish if pooled samples from the floor, shoe cover samples, and slurry tank samples capture antibiotic resistance at herd level to the same degree, a total of 5 stables were sampled during sampling 2 (Table 1). Individual animal sampling was not included as this stage, since the relation between individual and pooled samples were determined above. During sampling 2, 4 pens were randomly chosen in each stable and sampled (n = 20). The pen floor samples and shoe cover samples were collected in each stable as described above with the exception that 2 shoe cover samples were collected for each stable (pen floor samples n = 20; shoe cover samples n = 10) instead of 4. Furthermore, slurry tank samples were taken at 1 m, 1.5 m, and 2 m depths from 3 sampling spots that were spaced approximately 50 cm apart. One ml from each of the 3 depths was mixed for each corresponding sampling spot and homogenized by vortexing. Each slurry tank sample thus consisted of a pool of the 3 depths (denoted as slurry tank 1, slurry tank 2, and slurry tank 3; total n = 3).

All samples were collected in plastic containers with tight lids or sealed plastic bags and were placed in coolers immediately after sampling. They were then stored at 5°C until analysis in the laboratory the following day. Thereafter, all samples were frozen at -80°C.

### Sample Processing and Pooling

The non-sampled shoe cover weight was subtracted from the sample shoe cover weights, and defined amounts of PBS buffer were added to the sampled shoe covers. This dilution was corrected for in the statistical calculations. The shoe cover samples were then vigorously vortexed for 1 minute in order to extract as much sample from the shoe cover as possible. Thereafter, each shoe cover was “wrung out” to extract the remaining liquid. A 10^-1^ dilution was made from the resulting solutions by mixing 1 ml of the shoe cover sample solution with 9 ml PBS buffer.

Pooled samples were created in the laboratory as outlined in Table 1 and consisted of: 1) Pool of all individual animals from sampling 1 (All Animals), 2) Pools of animals within each pen (individual animal pool pen), 3) Pool of pen floor samples from pens 1–4 in each stable (pen floor pool stable), 4) Pool of the shoe cover samples in each stable (shoe cover pool stable), 5) Pool of pen floor samples from each stable (pen floor pool herd), 6) Pool of shoe cover samples from each stable (shoe cover pool herd), 7) Slurry tank samples (pool slurry). Before pooling the samples in the laboratory, a 10^-1^ dilution of the sample was made by suspending 1 g of feces in 9 ml PBS buffer. The pool samples were made by mixing 1 ml of the 10^-1^ resolution for each corresponding sample included in the pool for 1 minute.

### CFU counts of coliform bacteria

Coliform bacteria are often used as indicator bacteria in antibiotic resistance surveillance programs and were used in the current study [15,16]. The 6x6 drop plate procedure was used for CFU counts of coliform bacteria [17]. Briefly, ten-fold serial dilutions from each swine fecal sample collected were made. One drop (20 μl) of each dilution was carefully placed on MacConkey plates (Oxoid) without and with antibiotics (Ampicillin (16 mg l^-1^); Erythromycin (32 mg l^-1^); Sulfamethoxazole (256 mg l^-1^); Tetracycline (16 mg l^-1^)) and incubated at 37°C for 24 hours.

### DNA extraction for quantification by qPCR

DNA was extracted from the 10^-1^ dilutions of the swine fecal samples. Sample preparation consisted of homogenization of the 10^-1^ dilutions by vigorous vortexing with a 5 mm stainless steel bead (Qiagen, Copenhagen, Denmark). Thereafter, 350 μl of the 10^-1^ dilutions was transferred to a new eppendorf tube and put on ice. The samples were then lyzed for 1 minute (15 Hz at room temperature) in Tissuelyser II (Qiagen) followed by centrifugation for 90 seconds at 10000 rpm. The supernatant (300 μl) was transferred to a new eppendorf tube and 20 μl Proteinase K (20 mg/ml) (Promega, Roskilde, Denmark) was added (not on ice). The samples were then immediately loaded into the QiaSymphony robot using the QiaSymphony DSP Virus/Pathogen Mini Kit (Qiagen) according to the manufacturer’s instructions. The final elution volume was 85 μl.

Both a negative and positive DNA extraction control was run in parallel with the samples during each DNA extraction. The negative extraction control was water and was a control to test for sample contamination during the extraction process. The positive extraction control was included to ensure that antibiotic resistance gene levels were not affected by the DNA extraction process, and consisted of a modified pig feces sample positive for the majority of antibiotic resistance genes included in this study. The genes that were not present in this sample, defined as having a cycle number (Cq)>30 in qPCR, were spiked into the sample by adding 100 μl 10^5^ amplicons μl^-1^ to a final volume of 1500 μl extraction control. This corresponded to Cq values between 20 and 30.

### Primers and probes

Fourteen antibiotic resistance gene determinants that have previously been associated with swine manure (*tet*(-A,-B,-C,-M,-O,-W) [9,18–22], *ermB/F* [10,11,22], *sulI*/*sulII* [23,24], *vanA* [22], *bla*_CTX-M-1_ group [25], *bla*_CMY-2_], and *bla*_SHV_ family [11,26]) were selected. Other inclusion criteria for the antibiotic resistance genes included: The use of the antibiotic class in the Danish pig production, the association of the gene with PCR detection, and the occurrence of the gene in a wide bacterial population. It also had to be feasible to design a qPCR assay for the chosen genes utilizing the same temperature profile.

Sequences of the chosen antibiotic resistance determinant were retrieved from GenBank (during 2011–2012), and the sequences described in intestinal commensals and in pigs were chosen for alignment using ClustalX [27]. The conserved regions in the alignments were then used for primer and probe design using Primer3Plus Web Interface (Free Software Foundation, Boston, MA, USA). Previously published primers with annealing temperatures of 60–61°C and probes with annealing temperatures of approximately 70°C were used and modified if necessary (Table 2).

**Table 2:** PCR primer and probe sequences.

Primers	Gene target	Sequence (5’ 3’)	Ann. temp(°C)	Amplicon size (bp)	GenBank access. no.^a^	Reference
FP_TETA_2	*tet*(A)	TTGGCATTCTGCATTCACTC	60	125 (840–974)	X00006	This study
RP_TETA_2		GAAGGCAAGCAGGATGTAGC	60			
PR_TETA_2		GATCACCGGCCCTGTAGCCG				
FP_TETB_Aminov^b^	*tet*(B)	TTACGTGAATTTATTGCTTCGG	60	206 (913–1119)	EF646764	[28] and this study
RP_TETB_Aminov		ATACAGCATCCAAAGCGCAC	60			
PR_TETB_Aminov_own		CGCCGACCAAATCGGTCAGA				
FP_TETC_6	*tet*(C)	GCCAGTCACTATGGCGTGCT	60	120 (124–244)	EU751613	This study
RP_TETC_6		CAAGTAGCGAAGCGAGCAGG	60			
PR_TETC_6		ACTGTCCGACCGCTTTGGCC				
FP_TETM_7	*tet*(M)	CAACGAGGACGGATAATACGC	60	191 (119–311)	X92947	This study
RP_TETM_7		CCATCTTTTGCAGAAATCAGTAGA	60			
PR_TETM_7		GGTGAACATCATAGACACGCCAGGA				
FP_TETO_Böck	*tet*(O)	AAGAAAACAGGAGATTCCAAAACG	60	75 (607–682)	AY660531	[29]
RP_TETO_Böck		CGAGTCCCCAGATTGTTTTTAGC	60			
PR_TETO_Böck		ACGTTATTTCCCGTTATCACGGAAGCG				
FP_TETW_Smith	*tet*(W)	GCAGAGCGTGGTTCAGTCT	60	66 (411–476)	AJ222769	[30]
RP_TETW_Smith		GACACCGTCTGCTTGATGATAAT	60			
PR_TETW_Smith		TTCGGGATAAGCTCTCCGCCGA				
FP_SUL1_2	*sulI*	ACGAGATTGTGCGGTTCTTC	60	159 (440–598)	EU056266	This study
RP_SUL1_2		CCGACTTCAGCTTTTGAAGG	60			
PR_SUL1_2		ACCGGCTCATCCTCGATCCG				
FP_SUL2_3	*sulII*	GATATTCGCGGTTTTCCAGA	60	141 (313–453)	AY360321	This study
RP_SUL2_3		CGCAATGTGATCCATGATGT	60			
PR_SUL2_3		AAGACGGGCAGGCAGATCGG				
FP_ERMB_Böck	*ermB*	GGATTCTACAAGCGTACCTTGGA	60	86 (390–476)	AB563188	[29]
RP_ERMB_Böck^b^		TGGCAGCTTAAGCAATTGCT	60			
PR_ERMB_Böck		CACTAGGGTTGCTCTTGCACACTCAAGTC				
FP_ERMF_KNAPP	*ermF*	TCGTTTTACGGGTCAGCACTT	60	182 (24–205)	M14730;M17124;M17808;M62487	[11] and this study
RP_ERMF_KNAPP		CAACCAAAGCTGTGTCGTTT	60			
PR_ERMF_OWN		ATATTGGGGCAGGCAAGGGGTT				
FP_vanA_Böck	*vanA*	CTGTGAGGTCGGTTGTGCG	60	64 (614–705)	AF516335	[29]
RP_vanA_Böck		TTTGGTCCACCTCGCCA	60			
PR_vanA_Böck		CAACTAACGCGGCACTGTTTCCCAAT				
FW3_SHV_lahey	*bla*_SHV_ family	GCTGGAGCGAAAGATCCACT	60	247 (258–504)	All available at http://www.lahey.org/Studies/	[31] and this study
RV5_SHV_lahey		CGCCTCATTCAGTTCCGTTT	60			
Pr_SHV_Lahey2		AYGTCACCCGCCTTGACCGC				
FW3_CMY-2_Lahey	*bla*_CMY-2_	AGACGTTTAACGGCGTGTTG	60	127 (260–387)	All available at http://www.lahey.org/Studies/	[31] and this study
RV4_CMY-2_Lahey		TAAGTGCAGCAGGCGGATAC	60			
PR_CMY-2_Lahey		TATCGCCCGCGGCGAAAT				
FW_CTX-M-1	*bla*_CTX-M-1_group	ATGTGCAGYACCAGTAARGTKATGGC	58	335	X92506	[32]
RV_CTX-M-1		ATCACKCGGRTCGCCXGGRAT	58			
PR_CTX-M-1		CCCGACAGCTGGGAGACGAAACGT				
FW_SMI_114	16S rDNA	CGCGAAGAACCTTACC	60	126 (916–1041)	NA	The Public Health Agency of Sweden
R_SMI_115		ACTTAACCCAACATTTCAC	60			
PR_SMI_116		CACGAGCTGACGACAGCC				

Forward primer = FP; Reverse primer = RP; Probe = PR, gene targets, annealing temperatures (Ann. temp.), amplicon lengths in base pairs (bp), and GenBank sequence accession number (GenBank access. no.).

^a^ GenBank accession numbers for previously published primers and/or probes. For primers and probes designed in this study, a GenBank accession number representative of those included in the sequence alignments is given.

^b^ Primer modified to fit assay in the present study.

Potential primer and probe sequences were used to query GenBank DNA sequences using Basic Local Alignment Search Tool nucleotide (BLASTn) to determine specificity. The primer and probe sequences that matched the desired antibiotic resistant determinants were analyzed using Integrated DNA Technologies SciTools Oligoanalyzer (Integrated DNA Technologies, Inc., Coralville, IA, USA). Wherever necessary, degenerate bases were introduced into the primer/probe sequences to match all the sequences in the alignments. Primers (Table 2; S1 Table) were synthesized by TAG Copenhagen A/S (Frederiksberg, Denmark) and for 16S rDNA by DNA Technology A/S (Aarhus Denmark). Probes (Table 2) were synthesized by Applied Biosystems (Life Technologies, Naerum, Denmark).

The identity of all standard amplicons were verified by sequencing from both ends using the BigDye Terminator v3.1 Sequencing Kit on a 3130 Genetic sequencer (Applied Biosystems, Life Technologies). Due to the short amplicon lengths, sequencing attempts of the qPCR amplicon sequences failed. Therefore, the amplicon products were confirmed using the High Sensitivity DNA chip on an Agilent 2100 Bioanalyzer (Agilent Technologies, Walbronn, Germany). The qPCR assays were also tested for cross reaction by running each qPCR assay against a panel of 16 different antibiotic resistance gene amplicon negative controls at a concentration of 2x10^4^ copies μl^-1^.

### Generation of amplification standards

The standards used for absolute quantifications consisted of purified PCR amplicons from antibiotic resistance genes; this principle has successfully enabled absolute quantification of antibiotic resistance determinants previously [7,9,29]. The amplicon standards included in the present study were derived from bacterial strains or pig fecal samples (S2 Table). The bacterial strains positive for tetracycline and beta-lactam antibiotic resistance determinants were kindly provided by Yvonne Agersø (National Food Institute (DTU-FOOD), Lyngby, Denmark), those positive for sulphonamide resistance determinants and *ermB* by Anette M. Hammerum (Statens Serium Institut (SSI), Copenhagen, Denmark), the bacterial strain positive for *ermF* by Stefan Schwarz (Friedrich-Loeffler-Insitut (FLI) Neustadt-Mariensee, Germany), and the strain positive for *vanA* from Luca Guardabassi (Faculty of Medical and Health Sciences (SUND), University of Copenhagen). The fecal samples were provided by The Veterinary Institute, The Technical University (DTU-VET), Frederiksberg, Denmark.

Total DNA was extracted from the bacterial strains using Invitrogen-easy DNA kit (Invitrogen, Life Technologies, Naerum, Denmark) and from the fecal samples as described for the fecal samples collected for qPCR analysis. The amplicons were generated using the primers in S1 Table using a T3000 thermocycler for standard PCR (Biometra, Göttingen, Germany). The PCR Mastermix for *tet*(B), *tet*(C), *tet*(M), *tet*(O), *tet*(W), *sulI*, *sulII*, and *erm*B amplicon generation had the following concentrations per 25 μl reaction volume: 250 μM deoxynucleotide triphosphates (dNTPs), 1X buffer, 1.5 mM MgCl_2_, 0.5 μM of each forward (FW) and reverse (RV) primers, 1.25 U Platinum *Taq* DNA Polymerase (Invitrogen, Life Technologies, Naerum, Denmark) plus 2 μl DNA. Cycling conditions were: Initial denaturation for 4 min at 95°, followed by 30 cycles PCR, each cycle consisting of 15 sec at 94°C, 30 sec at 58°C and 60 sec at 72°C. There was a final extension for 5 min at 72°C.

The *tet*(A) and *ermF* amplicons were generated as previously described (*tet*(A) [33], *ermF* [11] using the following reaction mix: 25 μl reaction volume containing 250 μM dNTPs, 1X buffer, 1.50 mM MgCl_2_, 0.5 μM of each FW and RV primers, 1.25 U Platinum *Taq* DNA Polymerase (Applied Biosystems, Life Technologies) plus 2 μl DNA. *bla*_CTX-M-1_ group, *bla*_SHV_ family, *bla*_CMY-2_ and *vanA* amplicons were generated as previously described [26,34,35].

16S amplicons were generated as previously described [36] with the following modifications: The reaction mix contained 130 μg/ml of each FW and RV primer and 0.5 U Platinum *Taq* DNA Polymerase (Applied Biosystems, Life Technologies); the final elongation was extended to 5 min at 72°C.

Amplicon lengths were confirmed by gel electrophoresis and gene copy numbers were calculated after DNA quantification by UV spectrophotometry using a NanoDrop 3300 (Thermo Scientific, Wilmington, DE, USA). The qPCR standards were created by serially diluting the target gene amplicons in nuclease-free yeast tRNA (1:100 tRNA dilutions of 10 mg mL^-1^ (Applied Biosystems, Life Technologies)). The limit of quantification (LOQ) was defined as the lowest point in the amplicon standard serial dilution where all triplicates were positive. The limit of detection (LOD) was defined as the lowest concentration in the amplicon standard serial dilution where at least 1 of the triplicates was positive.

### Internal amplification control for qPCR assays

Internal control amplicons (ICA) consisting of lambda (λ) phage DNA flanked by the forward (FP) and reverse (RP) primer sequences of the respective antibiotic resistance genes were included in the respective qPCR assay as an internal amplification control (IC). The primers used were the antibiotic resistance qPCR assay primers (Table 2) with a λ phage DNA sequence added to the 3´end (extra sequences 5’-3’direction: FP ATGAATATGACCAGCCAAC, RP TTCACGCAGGGGAAATATCTTTC) [37]. The ICAs were generated on a T3000 thermocycler (Biometra, Göttingen, Germany) in a reaction volume of 50 μl with 50 μM MgCl_2_, 1X Buffer, 10 μM dNTPs, 50 μM of each forward and reverse primers, 1.25 U Platinum *Taq* DNA Polymerase (Applied Biosystems, Life Technologies), and 1 μL λ DNA (1 ng μl^-1^) (Applied Biosystems, Life Technologies). The cycling conditions were: 5 min incubation period at 94°C followed by 10X touchdown cycles from 58°C, each touchdown cycle consisting of 1 min at 94°C, 1 min at 58°C, and 1½ minute at 72°C. Thereafter, there were 5 cycles, each with 1 min at 94°C, 1 min at 48°C and 1½ min at 72°C followed by 8X touchdown cycling from 48°C, each touchdown cycle consisting of 1 min at 94°C, 1 min at 48°C and 1½ min at 72°C. Next there were 12 cycles, each with 1 min at 94°C, 1 min at 40°C, and 1½ min at 72°C. Finally, there was an elongation of 10 min at 72°C.

The ICA length of 690 bp was verified by gel electrophoresis and the ICA was serially diluted to 10^-11^. All antibiotic resistance gene qPCR assays were run on Rotorgene thermocyclers (Rotorgene Q-5plex and Rotorgene Q (Qiagen, Copenhagen, Denmark) with a Coxlam probe (VIC label 5’- CCACGAAGCCGCACFACTCCGC; Applied Biosystems, Life Technologies) and ICA added to the mastermix. In order to determine the concentration of ICA to use as an internal control the following was tested: 5 separate mastermixes with ICA PCR product dilutions within 10^-3^ to 10^-11^ and 1 “no internal control” were tested against the lowest 3 concentrations detectable by the respective antibiotic resistance gene qPCR assay. The ICA dilutions that did not inhibit the respective antibiotic resistance gene qPCR assays were used as the internal controls, where the λ DNA easy probe was detected on the yellow channel (530–555 nm) and the antibiotic resistance gene qPCR probes on the green channel (470–510 nm).

### Quantification of antibiotic resistance genes in pig fecal samples by qPCR

Quantitative PCR amplifications for the quantification of *tet*(A),*tet*(B), *tet*(C), *tet*(M), *tet*(O), *tet*(W), *ermF*, *ermB*, *sulI*, *sulII*, *bla*_CTX-M-1_ group, *bla*_CMY-2_, *bla*_SHV_ family, *vanA* and 16S in total DNA extracted from pig fecal samples were performed with Rotorgene thermocyclers (Rotorgene Q-5plex and Rotorgene Q, 72-well rotor 1–72) (Qiagen). The mastermixes are depicted in Table 3 and cycling conditions were: 10 min incubation period at 95°C followed by 45 cycles of PCR, each cycle consisting of 15 sec at 94°C and 30 sec at 60°C with a single fluorescence reading at green and yellow channels at the end of the extension stage. Each sample was tested in duplicate, along with a single point from the tenfold dilution series of the specific standard in triplicate, a single negative template control (NTC) that was 23μl mastermix and 2 μl water, and 1 positive and negative DNA extraction control. Quantification was performed using standard curves obtained from the PCR generated positive controls.

**Table 3:** Concentrations of reagents per reaction used in qPCR assays with a total reaction volume of 25 μl including 2 μl DNA.

Gene	qPCR Mastermix					Forward Primer	Reverse primer	Probe	Λ Probe	Λ PCR pro-duct
	Taqman Universal (Applied Biosystems)	Buffer	MgCl_2_mM	Platinum *Taq* Polymerase (Invitrogen, Life Technologies, Grand Island, NY, United States) U	dNTPs μM	μM	μM	μM	μM	ul
*tet*(A)	1X	-	-	-	-	0.5	0.5	0.2	0.2	1
*tet*(B)	_	1X	3.5	1.25	250	0.5	0.5	0.2	0.2	1
*tet*(C)	1X	-	-	-	-	0.5	0.5	0.2	0.2	1
*tet*(M)	-	1X	2.5	1.25	250	0.8	0.8	0.2	0.2	1
*tet*(O)	1X	-	-	-	-	0.6	0.6	0.2	0.2	1
*tet*(W)	1X	-	-	-	-	0.9	0.9	0.2	0.2	1
*ermB*	1X	-	-	-	-	0.5	0.5	0.2	0.2	1
*ermF*	-	1X	1.5	1.25	250	0.5	0.5	0.2	0.2	1
*sulI*	-	1X	1.5	1.25	250	0.8	0.8	0.2	0.2	1
*sulII*	1X	-	-	-	-	0.8	0.8	0.2	0.2	1
*vanA*	1X	-	-	-	-	0.6	0.6	0.2	0.2	1
*bla*_CTX-M-1_ group	-	1X	1.5	1.25	250	0.5	0.5	0.2	0.2	1
*bla*_CMY-2_	-	1X	3.0	1.25	250	0.6	0.6	0.4	0.2	1
*bla*_SHV_ family	-	1X	3.5	1.25	250	0.6	0.6	0.2	0.2	1
16S rDNA	1X	-	-	-	-	0.9	0.9	0.2	NA	NA

### The impact of pig fecal environment on quantification

The impact of pig fecal environment DNA on the quantification of the respective antibiotic genes, using the 14 antibiotic resistance gene qPCR assays and the 16S rDNA qPCR assay, was tested. Nuclease-free yeast tRNA amplicon serial dilutions were analyzed parallel to pig fecal DNA spiked with antibiotic resistance gene amplicon serial dilutions. Using serial dilutions of amplicons assures confidence of the number of templates that are added to start with when preparing the serial dilutions and assumptions of gene copy number per positive control bacteria are avoided, which may introduce bias if the bacteria lose their plasmid, have different plasmid copy numbers, or shed the antibiotic resistance gene during the extraction process [7].

### Statistical analysis

All figures and statistical tests were completed using R software (Version 3.0.1). The geometric mean of the technical replicates of gene copy numbers were used for further analysis. When only one of the technical replicates was above the LOQ than that value was used. Differences in gene copy numbers g^-1^ feces and in coliform bacteria CFU counts between pens from sampling 1 were calculated using Kruskal-Wallis rank sum test.

### Dataset information

The dataset is located at The National Veterinary Institute, Section for Bacteriology, Pathology and Parasitology, The Technical University of Denmark (latitude: 55° 41’ 4.34” N; longitude: 12° 32’46.65” E; elevation: 7.34 m).

## Results

### Accuracy of the qPCR assays

Standard curves for qPCR were generated using the serial dilutions of the amplification standards. The dynamic ranges of the antibiotic resistance gene assays were all linear over a measurement range >7 orders of magnitude and 5 orders of magnitude for the 16S rDNA assay (S3 Table). The amplicon standard serial dilutions were used for determining the linear dynamic range where R^2^ = 0.99, efficiency = [0.90; 1.10] and M≈-3.2. The efficiencies of the qPCR assays, determination coefficient (R^2^), dynamic range, quantification—and detection limits are all summarized in S3 Table.

The qPCR primers did not give unspecific reactions when tested against a panel of 16 different antibiotic resistance gene amplicon negative controls at 2x10^4^ copies μl^-1^. The amplicons of all specific qPCRs from fecal samples produced the expected specific gene sequence, without any non-specific amplification (data not shown).

The performance of the qPCR tests was further evaluated using extracted DNA from pig feces for spiking with antibiotic resistance genes. The efficiency remained between [0.90;1.10] and R^2^ above 0.99 for all assays except for *tet*(A) which showed an efficiency of 0.85, *tet*(M) with an efficiency of 0.84 and R^2^ = 0.87, *sulII* with an efficiency of 0.88, *vanA* with an efficiency of 0.87, and 16S rDNA with an efficiency of 0.84. Varying degrees of inhibition were observed when spiking the amplicons in pig fecal DNA extracts (1 to 4 Cq value increase), indicating a slight assay specific inhibition (data not shown). However, the dynamic ranges remained linear over a measurement range >4 orders magnitude in the spiked pig feces DNA environment (data not shown).

### Antibiotic resistant coliform CFU counts and qPCR gene copy number assessment in individual and pooled samples

**Fig 1:**
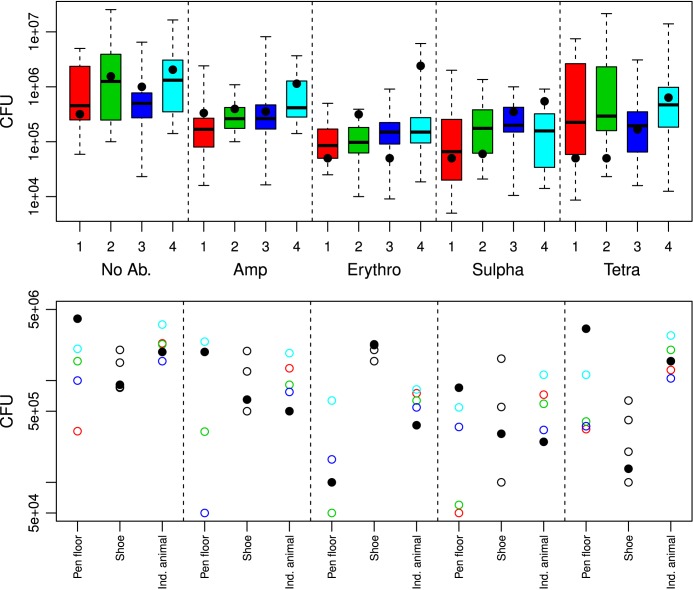
Sampling 1 coliform bacteria CFUs g^-1^ feces in individual animals within pens 1–4. Top: Boxplot showing the distribution of the coliform bacteria CFUs from sampling 1 in individual animals within pens 1–4 (pen 1, red, n = 22; pen 2, green, n = 20; pen 3, purple, n = 22; pen 4, blue, n = 20) on MacConkey plates (No Ab. = no antibiotics, Amp = ampicillin, Erythro = erythromycin, Sulpha = sulphonamide, Tetra = tetracycline). The bottom and top of the boxes are the first and third quartiles, respectively. The black band inside the box is the median and the “dotted-whiskers” represent the maximum (greatest CFU number values, excluding outliers) and minimum (lowest CFU value, excluding outliers). The solid circles are the individual animal pool pen samples within each pen. Bottom: The distribution of coliform bacteria CFUs from different sampling and pooling methods from sampling 1. The sampling methods are given under the bottom (Pen floor = pen floor sample; Shoe = shoe cover sample; Ind. animal = Individual animal sample). Each pen is represented by their colored circle (pen 1, red circle; pen 2, green circle; pen 3, purple circle; pen 4, blue circle), and the corresponding stable pools are solid black circles. The individual shoe cover samples are empty black circles, and the corresponding stable pool is a solid black circle.

CFU counts of coliform bacteria were chosen as an approach to antibiotic resistance estimation in the collected swine fecal samples. Fig 1 (top) shows a boxplot over the CFU counts of resistant coliform bacteria in the individual animals within the 4 pens from sampling 1. There was a significant difference between pens for the ampicillin resistant CFUs only (p<0.05; data not shown).

Next, the pooled samples of individual animals within pens (Fig 1, top, solid circles) were evaluated in relation to the antibiotic resistance levels in the non-pooled individual animal samples The individual animal pool pen samples were largely dispersed compared to the median of corresponding non-pooled individual animal samples. This was most prominent in the erythromycin, sulphonamide, and tetracycline groups (Fig 1, top).

Fig 1 (bottom) depicts the pen floor, shoe cover, and individual animal coliform CFU counts for each pen from sampling 1. The respective stable pools are also included (pen floor pool stable, shoe cover pool stable, and individual animal pool stable). There was a large variation within each sampling method with the pen floor samples having the largest range. The shoe cover and individual animal stable pools either lie among or below their corresponding pen pools, where the pen floor CFU counts were below the pen floor pool stable for all groups except ampicillin and erythromycin.

The *bla*_CTX-M-1_ group, *bla*_CMY-2_, *bla*_SHV_, and *vanA* antibiotic resistance genes were not detected in any samples and were therefore excluded from further analysis and all graphs. The tendencies described for the gene copy estimates in sampling 1 and 2 did not change after normalization with 16S rDNA (S1 and S2 Figs). Therefore, only the absolute quantifications by qPCR were used for further data analysis.

**Fig 2:**
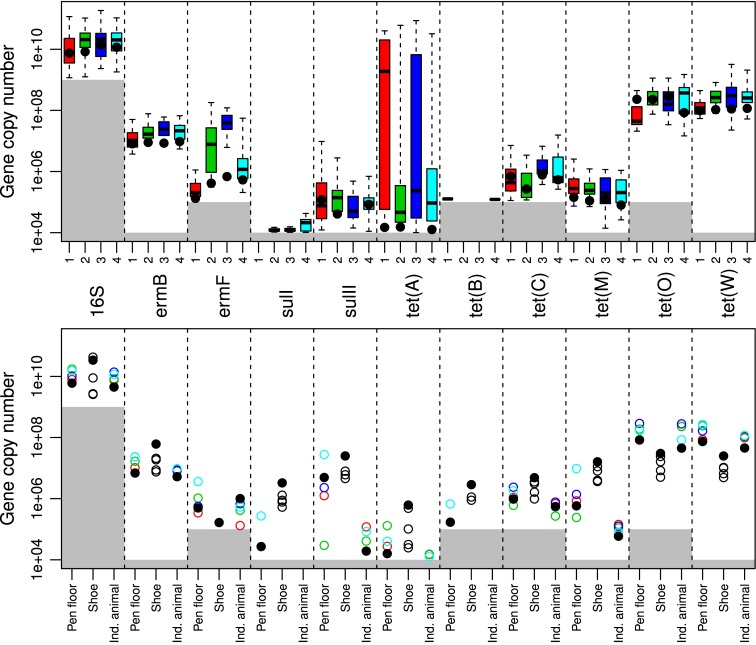
Sampling 1 gene copy numbers g^-1^ feces in individual animals within pens 1–4. Top: Boxplot showing the distribution of gene copies above the limit of quantification, LOQ (grey area = below LOQ) for individual animals within pens 1–4 from sampling 1 (pen 1, red, n = 22; pen 2, green, n = 20; pen 3, purple, n = 22; pen 4, blue, n = 20). The bottom and top of the boxes are the first and third quartiles, respectively. The black band inside the box is the median and the “dotted-whiskers” represent the maximum (greatest gene copy number values, excluding outliers) and minimum (least gene copy number value, excluding outliers). The solid circles are the individual animal pool pen samples within each pen. Each column represents a denoted gene (the respective genes are depicted in the middle of the figure and are shared for the top and bottom section of Fig 2). Bottom: The distribution of gene copies above the LOQ (grey area = below LOQ) for different sampling and pooling methods from sampling 1. The sampling methods are given under the bottom figure (Pen floor = pen floor sample; Shoe = shoe cover sample; Ind. animal = Individual animal sample). Each pen is represented by their colored circle for the pen floor and Ind. animal samples (pen 1, red circle; pen 2, green circle; pen 3, purple circle; pen 4, blue circle). The shoe cover samples are the same 4 shoe covers that were used in all 4 pens (individual shoe cover samples are empty black circles). The stable pools of the respective sampling method, pen floor, shoe cover, Ind. animal, are solid black circles.

Fig 2 (top) illustrates the copy number distribution of each gene for all animals sampled within each pen. Genes such as *sulII*, *ermB*, and *tet*(M) were relatively constant between pens while the genes *ermF*, *tet*(A), *tet*(C), *tet*(O), and *tet*(W) varied in at least 1 of the 4 sampled pens. Generally *ermB*, *ermF*, *tet*(O), and *tet*(W) had higher copy numbers g^-1^ feces compared to *sulI*, *sulII*, *tet*(A)-*tet*(C), and *tet*(M). *sulI* gene copy estimates were particularly low while *tet*(B) had levels below the LOQ in pens 2 and 3.

The gene copy numbers of *ermB*, *ermF*, *tet*(C), *tet*(O), and *tet*(W) were significantly different between pens (p<0.05 for *ermB*, *tet*(C), *tet*(W); p<0.0001 for *ermF* and *tet*(O)). Pen 1 consistently had lower gene copy number g^-1^ feces for *ermB*, *ermF*, *tet*(O), and *tet*(W) compared to pens 2–4 with *tet*(C) also having lower gene copy number g^-1^ feces in pen 2 (Fig 2 (top)). *ermF* in particular varied between pens with a large variation within pen 2. *tet*(A) generally had large variations within pens compared to the other genes with the highest levels in pens 1 and 3.

**Fig 3:**
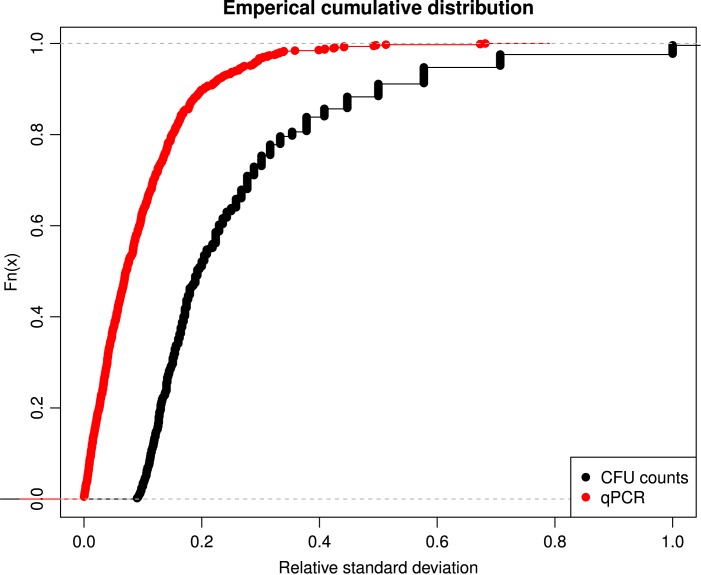
Relative standard deviations of the coliform CFU and qPCR gene copy number estimates g^-1^ feces. Empirical cumulative distribution (Fn(x)) plotted against the relative standard deviations of the CFU and gene copy number estimates illustrating the relationship between uncertainties of calculated estimates and the true laboratory determined estimates for CFU counts (black) and qPCR gene copy numbers (red), respectively.

Fig 3 illustrates the relative standard deviations of the fecal estimates of the coliform CFU counts and qPCR gene copy numbers. The qPCR gene copy numbers have lower relative standard deviations compared to the coliform CFU counts. The estimated relative standard deviation was under 20% for only 50% of the coliform CFU counts, while the relative standard deviation of qPCR gene copy numbers was under 20% for 90% of the cases. Therefore, only qPCR gene copy number g^-1^ feces estimates were used to assess the different sampling and pooling methods.

The next step was to establish whether pooled samples from the floor and shoe covers cold be used instead of sampling individual animals. Fig 2 (bottom) depicts the distribution of each gene within pens 1–4 for the pen floor samples, shoe cover samples, pen floor pool stable, shoe cover pool stable, individual animal pool pen, and all animals. For all genes except for *ermF* and *tet*(C), there was a tendency for lower gene copy number estimates in the laboratory pools of individual animals within each pen when comparing to the pen floor samples. The shoe cover stable pools were consistently higher than the non-pooled shoe cover samples. In contrast, there were no *ermF* individual shoe cover samples above the LOQ but there were positive individual animal samples in all four pens (Fig 2 bottom), however the shoe cover pool stable was positive.

### Pooling strategies at herd level

**Fig 4:**
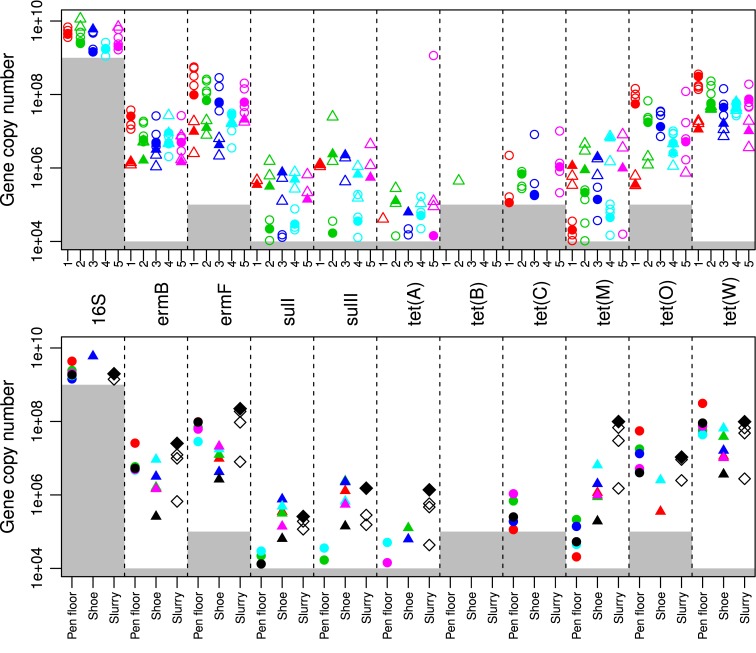
Sampling 2 gene copy numbers g^-1^ feces in sections 1–5. Top: Copy numbers of genes g^-1^ feces above the limit of quantification, LOQ (grey area = below LOQ) for pen floor samples (circles) and shoe cover samples (triangles) within sections 1–5 in sampling 2 (section 1, red; section 2, green; section 3, purple; section 4, blue; section 5, pink). The laboratory pooled samples are included for each section in their respective color (pen floor pool stable, solid circle; shoe cover pool stable, solid triangles). Each column represents a denoted gene (the respective genes are depicted in the middle of the figure and are shared for the top and bottom section of Fig 4). Bottom: Copy numbers of genes g^-1^ feces above the LOQ (LOQ = grey area) for pen floor pool stable samples (solid circles); shoe cover pool stable samples (solid triangles) for stables 1–5 (stable 1, red; stable 2, green; stable 3, purple; stable 4, blue; stable 5, pink); Slurry samples 1–3 (white diamond); Pen floor pool herd samples (black solid circle); Shoe cover pool herd sample (black solid triangle); Pool slurry (black solid diamond). The sampling methods are given under the bottom figure (Pen floor = pen floor pool herd; Shoe = shoe cover pool herd; Slurry sample).

The final step was to establish if pooled samples from the floor, shoe cover samples, and slurry tank samples captured antibiotic resistance at herd level to the same degree. A comparison of results from sampling 2 including pen floor samples and shoe cover samples is shown in Fig 4 (top) together with the pooled samples from each category. Each section had up to 4 pen floor samples (1 from each pen) and 2 shoe cover samples with their respective laboratory pools. For *ermB*, *ermF*, *tet*(C), *tet*(O), and *tet*(W) the shoe cover samples gave lower estimates compared to the pen floor samples with the pools following the same pattern. For *sulI*, *sulII*, *tet*(A), *tet*(B), and *tet*(M), however, the shoe cover samples were higher than the pen floor samples. The 16S rDNA levels were stable regardless of the sampling and/or pooling methods for sampling 1 and sampling 2 (Figs 2 and 4).

Fig 4 (bottom) compares the pen floor and shoe cover pools both at stable and herd levels. The pooled and individual slurry samples from sampling 2 are also depicted in Fig 4 (bottom). The assays that had shoe cover samples with higher levels than the pen floor samples within stables 1–5 from sampling 2 (*sulI*, *sulII*, *tet*(A), and *tet*(M) (Fig 4 top)) also had higher shoe cover samples in the corresponding stable and herd level pools (Fig 4 bottom). The slurry tank samples complemented the pen floor and shoe cover herd pools, although it appeared that 1 of the 3 slurry tank samples was consistently lower than the others for all genes. Furthermore, *sulII* became negative at the pen floor herd pool despite having positive values in 2 out of 5 stable pools, and *tet*(A) had negative pen floor herd pools for and shoe cover herd pools despite their positive stable pools. *tet*(B) had a single positive shoe cover sample in stable 2 (Fig 4 top) with no positive results for stable pools, herd pools, and slurry tank samples (Fig 4 bottom). *tet*(C) had positive pen floor samples and pen floor pool stable samples in all stables except stable 4 (Fig 4 top). The *tet*(C) pen floor pool herd sample was positive (Fig 4 bottom), but the shoe cover and slurry tank samples were all negative (Fig 4 top and bottom).

## Discussion

Monitoring the antibiotic resistance patterns of infectious bacteria and their distribution aids disease prevention and control. Quantification of antibiotic resistance levels facilitates antibiotic resistance surveillance, ultimately helping to contain and prevent infections caused by antibiotic resistant bacteria. In the present study, 14 qPCR assays quantifying antibiotic resistant determinants and coliform bacteria CFU counts were used as two different approaches to antibiotic resistance estimation in swine fecal samples. This was done by applying both methods to swine fecal samples collected using different sampling and pooling methods. The main findings of our research were that the qPCR method detected significant differences in antibiotic resistance where the coliform CFU counts showed no significance, and furthermore, qPCR gene copy estimates in swine feces had reduced relative standard deviations compared to coliform CFU counts in the same samples, and therefore differences in antibiotic resistance levels between samples were more readily detected. This implicates that qPCR was a good representative for the general resistance level.

### Accuracy of the qPCR assays

Primer design, DNA quality, thermal profile, and mastermix all play an important role in the specificity and quality of the PCR product, and sensitivity of the assay [38]. qPCR optimization is therefore required for each untested assay, with subsequent verification of assay specificity. In the current study, there was no cross reaction when each assay was tested against a panel of 16 different antibiotic resistance gene amplicons negative controls. Furthermore, the amplicons of all specific qPCRs from fecal samples produced the expected specific gene sequence, without any non-specific amplification (data not shown). The design of qPCR assays containing internal, positive- and negative-extraction controls ensured a thorough PCR inhibition and DNA extraction procedure control.

Assay inhibition in the spiked pig fecal DNA samples compared to water was seen as slightly lower efficiencies and higher corresponding Cq values in the spiked fecal DNA samples. This discrepancy between DNA from complex environmental samples has been described and was expected [39,40]. The dynamic range remained linear with a minimum 4-fold magnitude in the spiked pig fecal DNA samples for all assays (data not shown) which is sufficient for genomic DNA, and R^2^ remained above 0.99 in all but 1 assay. Therefore, the slight variation seen in the spiked fecal DNA samples compared to sterile water is not considered to notably alter the assays’ performance in quantifying antibiotic resistance genes in pig fecal samples. We believe that the DNA extraction protocol, primer sets, and corresponding probes possess the characteristics necessary for application to antibiotic resistance gene quantification in pig fecal samples.

### Antibiotic resistant coliform CFU counts and qPCR gene copy number assessment in individual and pooled samples

When observing the coliform CFU counts in individual animals between pens, only a single group (ampicillin) showed a significant difference in resistant coliform bacteria. In contrast, significance was found in qPCR gene copy estimates for erythromycin and tetracycline, while no beta-lactamase genes were detected emphasizing how the two methods used for antibiotic resistance estimation yield each their endpoint. The phenotypic ampicillin resistance could be due to other ampicillin resistance encoding genes than those included in the present study emphasizing a key limit to the qPCR method, namely not all antibiotic resistance genes are included. In contrast, the phenotypic CFU counts are limited to coliforms representing only a fraction of the intestinal bacterial population, and may lead to underestimates of the true antibiotic resistance levels due to the limitations of the chosen indicator bacteria. Therefore, when monitoring antibiotic resistance, we argue that it is favorable to quantify the genes by qPCR instead of relying on phenotypic determination. This ensures that the entire bacterial population is represented while the denoted genes of interest (and therefore resistance) are also included. This principle is illustrated when assessing the coliform bacterial CFU counts in individual animals (Fig 1 top). Here, the erythromycin group had some of the lowest CFU counts overall. This contradicts the gene copy numbers g^-1^ feces for the corresponding *ermB* and *ermF* genes which are 3^rd^ and 4^th^ highest after *tet*(O) and *tet*(W) (Figs 2 and 4). A tentative conclusion from this is that the *ermB* and *ermF* genes reside in bacteria found in the intestines other than coliform bacteria as suggested by [31,41–46].

The CFU estimates of the individual animal pool pen samples were found not to represent an average of the non-pooled individual animal samples. This means that, at pen levels, the pooled samples from individual animals were not representative for the individual animals. There were also variations when comparing different sampling methods between pens (Fig 1, bottom) making it difficult to find differences between pens using coliform CFU counts. This is likely due to the large relative standard deviation found for each coliform CFU estimate (Fig 3). Therefore, only qPCR gene copy number g^-1^ feces were used to assess the different sampling and pooling methods.

The qPCR assays revealed interesting differences when assessing whether sampling and pooling strategies within a pig stable were representative of individual animal sampling. Pen 1 consistently had lower gene copy estimates for *ermB*, *ermF*, *tet*(O), and *tet*(W) compared to pens 2–4 with *tet*(C) also having lower estimates in pen 2 (Fig 2 top). These differences in gene copy number g^-1^ feces between pens were found statistically significant, and could mean that the antibiotic resistance genes do not easily spread between pens. The apparent variation in *tet*(A) estimates seen in Fig 2 (top) in pens 1 and 3 is caused by the graph only illustrating results from positive animals. Thus, few animals had high gene copy numbers (above 1x10^6^), where for half of them, one of the technical replicates was below the LOQ and therefore had no effect on the graph. This was solely seen for *tet*(A).

Bibbal et al. [39] monitored the *bla*_TEM_ excretion in pigs and found that the fecal excretion of *bla*_TEM_ genes showed large, individual day-to-day fluctuations [39]. Similar fluctuations in gene excretion could account for variations when quantifying antibiotic resistance genes. Antibiotic resistance is dynamic as its spread and maintenance is subject to fluctuations in host organism migration and/or persistence, antibiotic gene migration, and presence of selection pressure [40]. When collecting rectal samples at a single time point, the level found in all the individual animals within a pen may depend on the time a single animal within the pen has excreted the specific gene. The individual animal pool pen samples are more uniform than the individual animal sampling, as all of the animals and their respective antibiotic resistance levels are represented (Fig 2, top). If a single fecal sample with high levels of antibiotic resistance levels is included in a pool, it will mask the samples containing lower gene levels. On the other hand, if there also are sufficient fecal samples with low gene levels then they will dilute the high level sample [47].

For the majority of the genes, the individual animal pen pool resulted in lower gene copy number estimates when compared to the pen floor samples for each corresponding pen (Fig 2 and S1 Fig). This could be due to the dilution effect of the increased volume in the individual animal pen pools which were composed from >19 samples compared to the pen floor samples that consisted of 5 individual samples [48]. Furthermore, pen floor samples are collected from older feces that has resided on the ground permitting liquid evaporation and run off from the feces. This may result in a higher concentration of resistance genes in the sampled portion of the pen floor fecal sample and can be an advantage when quantifying low prevalence genes. This is illustrated by the fact that *tet*(B) was below the LOQ for the individual animal samples in 50% of the pens, while the pen floor sample from pen 4 and the pen floor pool stable samples were both positive for *tet*(B). In contrast, the individual shoe cover samples were negative for *ermF* while the shoe cover pool stable and individual animal samples from pens 1–4 were positive. Overall, this indicates that the shoe cover pool stable and the pen floor pool stable samples may be used instead of sampling individual animals.

### Pooling strategies at herd level

During sampling 2, 5 stables were sampled from the same herd as sampling 1. An interesting observation was that, for some genes the shoe cover samples were lower than the pen floor samples (*ermB*, *ermF*, *tet*(O), *tet*(W)) but were higher for *sulI*, *sulII*, *tet*(A), and *tet*(M). This was observed both in sampling 1 (Fig 2 bottom) and the stable and herd pools from sampling 2 (Fig 4). The shoe cover samples varied in how much roughage they collected. Thus, if the shoe cover samples with lower gene copy number estimates had more roughage, the sample would weigh more without the entire weight being attributable to feces. Consequently, this could result in low gene copy number g^-1^ feces. In contrast, the shoe cover with high gene copy number estimates suggest that the antibiotic resistance genes represented in the sample may depend on which sampling method is used, as different bacteria harbor antibiotic resistance genes while residing in a specific fecal fraction. The gastrointestinal tract is a complex ecosystem containing at least 400 different bacterial species residing in regional habitats [49]. Hence, the shoe cover samples may be capable of collecting fractions of feces that pen floor or individual fecal samples cannot as the shoe cover samples were both saturated with liquid and covered with feces after sample collection. Furthermore, the entire pen floor is covered during shoe cover sampling thus increasing the likelihood of collecting a fecal sample positive for a given gene.

Several genes tested positive in at least one non-pooled sample type which then turned negative after pooling (sampling 1 *sulI*, *sulII*, *tet*(A); sampling 2 *tet*(A), *tet*(B), *tet*(C), *sulII*). Pooling may increase the risk of a sample becoming negative, especially if the gene copy number g^-1^ feces initially are relatively low. If there are sufficient numbers of negative samples included in the pool then the low prevalence gene concentration is diluted potentially resulting in levels under the assay’s quantification limit [48]. The sensitivity of a specific assay is therefore dependent on the gene prevalence, the number of samples included in the pool, the gene concentration in samples collected from positive animals, and the quantification limit of the assay [47,48]. A balance in the mentioned parameters could explain the apparent consistency in gene copy number g^-1^ feces between slurry tank samples, pen floor and shoe cover herd pools for the majority of the assays.

When choosing a sampling method for antibiotic resistance determination at herd level by qPCR, we recommend either pen floor samples or shoe cover sampling. Both sampling types were able to quantify antibiotic resistance genes in swine feces. Pen floor samples were easily attainable and are representative when pooled at the stable level, but several were negative when pooled at herd level (Fig 4 bottom). In contrast, the shoe cover samples include the entire pen, thus representing more animals and increasing the likelihood of finding antibiotic resistant determinants when present. Furthermore, fecal fractions not represented in pen floor samples and individual animal samples may be represented in the shoe cover samples. However, the shoe cover samples should not be pooled as the herd pools were negative for several of the assays. The slurry tank samples were also promising; their gene copy levels were consistent with those quantified in the pen floor and shoe cover samples. The slurry tank contains feces from the entire herd from a time period of approximately 6 months and may therefore give a better estimate of the herd antibiotic resistance levels. Further studies should be conducted where a series of slurry tank samples are collected from several pig herds over a longer time period, for example 6 months, in order to clarify the dynamics of antibiotic resistance genes in slurry tanks.

This study has utilized two different approaches to antibiotic resistance surveillance to assess different sampling and pooling methods of swine fecal sample collection that yielded two different endpoints, namely 14 qPCR assays to quantify antibiotic resistance genes in swine feces and CFU counts of coliform bacteria in the same samples. To our knowledge, this is the first study that tests sampling and pooling strategies for antibiotic resistance surveillance using qPCR determination of antibiotic resistance in total DNA extracted from swine feces [12,50,51]. It is necessary to understand the quantified antibiotic resistance gene levels in order to define if certain levels pose a potential risk or if they represent the specific farm. In order to do so, studies including parallel analysis of antibiotic resistance using several methods should be conducted across several pig farms.

Our results indicate that there is a great deal of variation in the antibiotic gene abundance within individual animals, pens, stables, and herds regardless of the sampling method. This variation could be systematically evaluated in greater detail using pen floor and/or shoe covering sampling methods supplemented with parallel slurry tank sampling.

## Supporting Information

S1 Dataset qPCR data sampling 1. (XLS)

S2 Dataset qPCR data sampling 2. (XLSX)

S3 Dataset Coliform CFU data sampling 1 and sampling 2. (XLSX)

S4 Dataset p-values sampling 1 significance for CFU and qPCR estimates. (XLSX)

S1 Fig Sampling 1 16S normalization of gene copy numbers g^-1^ feces. Top: Boxplot showing the distribution of gene copies normalized by 16S for individual animals within pens 1–4 from sampling 1 (pen 1, red, n = 22; pen 2, green, n = 20; pen 3, purple, n = 22; pen 4, blue, n = 20). The bottom and top of the boxes are the first and third quartiles, respectively. The black band inside the box is the median where the “dotted whiskers” represent the maximum (greatest relative gene copy values, excluding outliers) and minimum (least relative gene copy value, excluding outliers). The solid circles are the individual animal pool pen samples within each pen. Each column represents a denoted gene (the respective genes are depicted in the middle of the figure and are shared for the top and bottom section of S1 Fig). Bottom: The distribution of gene copies normalized by 16S for different sampling and pooling methods from sampling 1. The sampling methods are given under the bottom figure (Pen floor = pen floor sample; Shoe = shoe cover sample; Lab = Individual animal sample). The pens are each their colored circle (pen 1, red circle; pen 2, green circle; pen 3, purple circle; pen 4, blue circle), and the corresponding stable pools are solid black circles.
(EPS)

S2 Fig Sampling 2 16S normalization of gene copy numbers g^-1^ feces. Top: Copy numbers of genes normalized by 16S for pen floor samples (circles) and shoe cover samples (triangles) within stables 1–5 (stable 1, red; stable 2, green; stable 3, purple; stable 4, blue; stable 5, pink). The laboratory pooled samples are included for each stable in their respective color (pen floor pool stable, solid circle; shoe cover pool stable, solid triangles). Each column represents a denoted gene (the respective genes are depicted in the middle of the figure and are shared for the top and bottom section of S2 Fig). Bottom: Copy numbers of genes normalized by 16S for pen floor pool stable samples (solid circles); shoe cover pool stable samples (solid triangles) for stables 1–5 (stable 1, red; stable 2, green; stable 3, purple; stable 4, blue; stable 5, pink); Slurry samples 1–3 (white diamond); Pen floor pool herd samples (black solid circle); Shoe cover pool herd sample (black solid triangle); Pool slurry (black solid diamond). The sampling methods are given under the bottom figure (Pen floor = pen floor pool herd; Shoe = shoe cover pool herd; Slurry sample).
(EPS)

S1 Table Primers used to generate standard amplicons (forward primer = FP, reverse primer = RV). The amplicon size is in number of base pairs.
(PDF)

S2 Table Positive controls including bacterial isolates and fecal derived positive controls. (PDF)

S3 Table Efficiency, determination coefficient (R^2^), dynamic range, LOQ and LOD for qPCR assays. (PDF)
