# Supplementary material for: Sampling and Pooling Methods for Capturing Herd Level Antibiotic Resistance in Swine Feces using qPCR and CFU Approaches
Source: PLoS One. 2015 Jun 26;10(6):e0131672. doi: 10.1371/journal.pone.0131672 (PMC4483237; doi:10.1371/journal.pone.0131672)
Supplement: S1 Table — The amplicon size is in number of base pairs. (PDF) [file pone.0131672.s007.pdf]

S1 Primers used to generate standard amplicons (forward primer=FP, reverse primer=RV). The amplicon size is in number of base pairs (bp)

Primer	Target gene	Base sequence 5'-3'	Amplicon size (bp)	Reference
tet(A)_std_FW<br>*tet*(A)_std_RV	*tet*(A)	GTAATTCTGAGCACTGTCGC<br>CTGTCCTGGACAACATTGCTT	937	(1-3)
tet(B)_std_FW<br>*tet*(B)_std_RV	*tet*(B)	ACTTGTCTCCTGTTTACTCCCCTGAGC<br>GCCTTATCATGCCAGTCTTGCCAACG	1142	This study
tet(C)_std_FW<br>*tet*(C)_std_RV	*tet*(C)	CGCTCATCGTCATCCTCGGCAC<br>CAACCCGTTCCATGTGCTCGC	1091	This study
tet(M)_std_FW<br>*tet*(M)_std_RV	*tet*(M)	GAAGCGTGGACAAAGGTACAACGAGG<br>CGACGGGGCTGGCAAACAGG	1768	This study
tet(O)_std_FW<br>*tet*(O)_std_RV	*tet*(O)	CTGGCTCACGTTGACGCAGGAAAG<br>TATTCGGGCGGCGGGGTTG	1852	This study
tet(W)_std_FW<br>*tet*(W)_std_RV	*tet*(W)	AAAGACGACCTTGACGGAGAGCC<br>TCTCGCCAGTAAAGACAACTTCATCC	1697	This study
*sulI*_std_FW<br>*sulI*_std_RV	*sulI*	ATGGTGACGGTGTTCGGCATTCTG<br>GATCTAACCCTCGGTCTCTGGCGT	832	This study
*sulII*_std_FW<br>*sulII*_std_RV	*sulII*	CGGCATCGTCAACATAACCTCGGAC<br>CGGCTCGTGTGTGCGGATGAAG	730	This study
*ermB*_std_FW<br>*ermB*_std_RV	*ermB*	GGGCATTTAACGACGAAACTGGCT<br>ACTTTGGCGTGTTTCATTGCTTGATG	539	This study
*ermF*_std_FW<br>*ermF*_std_RV	*ermF*	TCGTTTTACGGGTCAGCACTT<br>CAACCAAAGCTGTGTCGTTT	182	(4)
*vanA*_std_FW<br>*vanA*_std_RV	*vanA*	GAAATCAACCATGTTGATGTAGCA<br>TTTGCCGTTTCCTGTATCCGT	572	(5)
CMY-2_std_FW<br>CMY-2_std_RV	bla_{CMY-2}	ATGATGAAAAAATCGTTATGC<br>GCTTTTCAAGAATGCGCCAGG	758	(6-8)

FW_SHV_OS5<br>FW_SHV_OS6	*bla*$_{SHV}$<br>family	TTATCTCCCTGTTAGCCACC<br>GATTTGCTGATTTCGCTCGG	854	(6-8)
CTX-M-U1_FW<br>CTXXM-U2nds_RV	*bla*$_{CTX\text{-}M\text{-}1}$<br>group	ATGTGCAGYACCAGTAARGTKATGGC<br>GGGTRAARTARGTSACCAGAAYSAGCGG	593	(6,8)
8FX<br>1407RX	16S rDNA	AGAGTTTGATCCTGGCTNAG<br>TGACGGGCGGTGTGTACAA	1392	(9)

References

(1) Guardabassi, L., Dijkshoorn, L., Collard, J. M., Olsen, J. E. and Dalsgaard, A. Distribution and in-vitro transfer of tetracycline resistance determinants in clinical and aquatic *Acinetobacter* strains. J Med Microbiol 2000; 49: 929-936.

(2) Saenz, Y., Brinas, L., Domínguez, E., Ruiz, J., Zarazaga, M., Vila, J. and Torres, C. Mechanisms of resistance in multiple-antibiotic-Resistant *Escherichia coli* strains of human, animal, and food origins. Antimicrob Agents Chemother 2004; 48: 3996-4001.

(3) Costa, D., Poeta, P., Saenz,Y., Coelho, A. C., Matos, M., Vinue, L., Rodrigues, J. and Torres, C. Prevalence of antimicrobial resistance and resistance genes in faecal *Escherichia coli* isolates recovered from healthy pets. Vet Microbiol 2008; 127: 97-105.

(4) Knapp, C. W., Zhang, W., Sturm, B. S. M. and Graham, D. W. (2010) Differential fate of erythromycin and beta-lactam resistance genes from swine lagoon waste under different aquatic conditions. Environ Poll 2010; 158: 1506-1512.

(5) Boeckelmann, U., Doerries, H. H., Ayuso-Gabella, M. N., Salgot de Marcay, M., Tandoi, V., Levantesi, C., Masciopinto, C., Van Houtte, E., Szewzyk, U., Wintgens, T. and Grohmann, E. Quantitative PCR monitoring of antibiotic resistance genes and bacterial pathogens in three Eurpean artificial groundwater recharge systems. Appl Environ Microbiol 2009; 75: 154-163.

(6) Hasman, H., Mevius, D., Veldman, K., Olesen, I. and Aarestrup, F. M. Beta-lactamases among extended-spectrum beta-lactamase (ESBL)-resistant *Salmonella* from poultry, poultry products and human patients in the Netherlands. J Antimicrob Chemother 2005; 56: 115-121.

(7) Archambault, M., Petrov, P., Hendriksen, R. S., Asseva, G., Bangtrakulnonth, A., Hasman, H. and Aarestrup, F. M. Molecular characterization and occurrence of extended-spectrum beta-lactamase resistance genes among *Salmonella enterica* serovar corvallis from Thailand, Bulgaria, and Denmark. Microb Drug Resist 2006; 12: 192-198.

(8) Agersø,Y., Aarestrup, F. M., Pedersen, K., Seyfarth, A. M., Struve, T. and Hasman, H. Prevalence of extended-spectrum cephalosporinase (ESC)-producing *Escherichia coli* in Danish slaughter pigs and retail meat identified by selective enrichment and association with cephalosporin usage. J Antimicrob Chemother 2012; 67: 582-588.

(9) Angen, Ø., Ahrens, P. and Tegtmeier, C. Development of a PCR test for identification of *Haemophilus somnus* in pure and mixed cultures. Vet Microbiol 1998; 63: 39-48.
