# Supplementary material for: Sampling and Pooling Methods for Capturing Herd Level Antibiotic Resistance in Swine Feces using qPCR and CFU Approaches
Source: PLoS One. 2015 Jun 26;10(6):e0131672. doi: 10.1371/journal.pone.0131672 (PMC4483237; doi:10.1371/journal.pone.0131672)
Supplement: S2 Table — (PDF) [file pone.0131672.s008.pdf]

S2 Positive controls including bacterial isolates and fecal derived positive controls.

Species	Name	Genes	Relevant resistance	Provider
NA	NA	*tet*(A)	Tetracycline	PCR amplification from swine fecal sample
E.coli	tetB, CSH50:Tn10	*tet*(B)	Tetracycline	Yvonne Agersø (DTU-Food)
E.coli	tetC, D07 pBR 322, Tet	*tet*(C)	Tetracycline	Yvonne Agersø (DTU-Food)
E.coli	tetM HB 101	*tet*(M)	Tetracycline	Yvonne Agersø (DTU-Food)
E.coli	tetO HB 101	*tet*(O)	Tetracycline	Yvonne Agersø (DTU-Food)
E.coli	tetW HB 101 pTnB1230, pUTetWup3	*tet*(W)	Tetracycline	Yvonne Agersø (DTU-Food)
E.coli	sul1, NCTC 50001	*sulI*	Sulphonamide	Anette M. Hammerum Statens Serum Institute(SSI)
E.coli	sul2, NCTC 50020	*sulII*	Sulphonamide	Anette M. Hammerum (SSI)
E.faecalis	ermB, D516C1 pAD2	*ermB*	MLS	Anette M. Hammerum (SSI)
E.coli	ermF, Tn4551, original plasmid pFD292 cloned into pUC19 (is ampicillin resistant)	*ermF* (0,7KB)	MLS	Stefan Schwarz (FLI)
E.coli	CTX-M-1,O149 77-30108-11	$bla_{\text{CTX-M-1}}$ group	ESBL	Yvonne Agersø (DTU-Food)
E.coli	CMY-2,F1 from ESC 1009 data	$bla_{\text{CMY-2}}$	AmpC Cephalosporinase	Yvonne Agersø (DTU-Food)
E.coli	SHV-12,F21 from ESC2009 data	bla_{SHV} family	ESBL	Yvonne Agersø (DTU-Food)
E. faecium	vanA, BM4147	*vanA*	Vancomycin	Luca Guardabassi (SUND)
NA	NA	16S rDNA	NA	PCR amplification from swine fecal sample
