# Supplementary material for: Sampling and Pooling Methods for Capturing Herd Level Antibiotic Resistance in Swine Feces using qPCR and CFU Approaches
Source: PLoS One. 2015 Jun 26;10(6):e0131672. doi: 10.1371/journal.pone.0131672 (PMC4483237; doi:10.1371/journal.pone.0131672)
Supplement: S3 Table — (PDF) [file pone.0131672.s009.pdf]

S3 Efficiency, determination coefficient (R^2), dynamic range, LOQ and LOD for qPCR assays.

Assay	Efficiency	R^2	Dynamic range copies reaction^{-1}	LOQ copies g^{-1} feces	LOD copies g^{-1} feces
tet(A)	0.92	0.99781	$1x10^9$-$1x10^1$	$1x10^4$	$1x10^3$
tet(B)	0.93	0.99942	$1x10^8$-$1x10^2$	$1x10^5$	$1x10^5$
tet(C)	0.96	0.99938	$1x10^8$-$1x10^1$	$1x10^5$	$1x10^3$
tet(M)	1.02	0.99690	$1x10^8$-$1x10^1$	$1x10^4$	$1x10^1$
tet(O)	1.13	0.99875	$1x10^8$-$1x10^2$	$1x10^5$	$1x10^1$
tet(W)	0.89	0.99908	$1x10^8$-$1x10^1$	$1x10^4$	$1x10^1$
ermB	0.91	0.99909	$1x10^9$-$1x10^1$	$1x10^4$	$1x10^2$
ermF	0.94	0.99587	$1x10^8$-$1x10^2$	$1x10^5$	$1x10^4$
sulI	1.03	0.99745	$1x10^8$-$1x10^1$	$1x10^4$	$1x10^3$
sulII	0.92	0.99845	$1x10^8$-$1x10^1$	$1x10^4$	$1x10^3$
vanA	0.78	0.99891	$1x10^8$-$1x10^1$	$1x10^4$	$1x10^3$
$bla_{CTX\text{-}M\text{-}1}$ group	0.98	0.99673	$1x10^9$-$1x10^1$	$1x10^4$	$1x10^2$
$bla_{CMY\text{-}2}$	0.96	0.99697	$1x10^8$-$1x10^2$	$1x10^5$	$1x10^4$
bla_{SHV} family	0.96	0.99791	$1x10^8$-$1x10^1$	$1x10^4$	$1x10^3$
16S rDNA	0.64	0.99045	$1x10^9$-$1x10^5$	$1x10^8$	NA
